# First Generic Teriparatide: Structural and Biological Sameness to Its Reference Medicinal Product

**DOI:** 10.3390/pharmaceutics16040537

**Published:** 2024-04-13

**Authors:** Jimena Fernández-Carneado, Mariona Vallès-Miret, Sílvia Arrastia-Casado, Ana Almazán-Moga, Maria J. Macias, Pau Martin-Malpartida, Marta Vilaseca, Mireia Díaz-Lobo, Mayte Vazquez, Rosa M. Sanahuja, Gemma Gambús, Berta Ponsati

**Affiliations:** 1 BCN Peptides SA, 08777 Barcelona, Spain; mvalles@bcnpeptides.com (M.V.-M.); sarrastia@bcnpeptides.com (S.A.-C.); aalmazan@bcnpeptides.com (A.A.-M.); bponsati@bcnpeptides.com (B.P.); 2 Institució Catalana de Recerca i Estudis Avançats (ICREA), 08010 Barcelona, Spain; maria.macias@irbbarcelona.org; 3 Institute for Research in Biomedicine (IRB Barcelona), The Barcelona Institute of Science and Technology (BIST), Baldiri Reixac 10, 08028 Barcelona, Spain; pau.martin@irbbarcelona.org (P.M.-M.); marta.vilaseca@irbbarcelona.org (M.V.); mireia.diaz@irbbarcelona.org (M.D.-L.); 4 GP-Pharm SA, 08777 Barcelona, Spain; mvazquez@gp-pharm.com (M.V.); rsanahuja@gp-pharm.com (R.M.S.); ggambus@gp-pharm.com (G.G.)

**Keywords:** teriparatide, sameness, comparative study, synthetic peptide, generic drug, peptide characterization

## Abstract

Teriparatide is an anabolic peptide drug indicated for the treatment of osteoporosis. Recombinant teriparatide was first approved in 2002 and has since been followed by patent-free alternatives under biosimilar or hybrid regulatory application. The aim of this study is to demonstrate the essential similarity between synthetic teriparatide BGW and the reference medicinal product (RMP), and thus to ensure the development of the first generic teriparatide drug. Hence, an extensive side-by-side comparative exercise, focusing on structural and biological activity, was performed using a wide range of state-of-the-art orthogonal methods. Nuclear magnetic resonance (NMR), ion mobility–mass spectrometry (IM–MS), UV, circular dichroism (CD) and Fourier transform infrared (FTIR) demonstrated the structural similarity between teriparatide BGW and the RMP. Comparative cell-based bioassays showed that the synthetic and recombinant peptides have identical behaviors. Teriparatide BGW, as a generic drug, provides an available treatment option for patients with osteoporosis and offers clinical benefits identical to those provided by the RMP.

## 1. Introduction

In a physiological setting, bone is continuously broken down and replaced—a remodeling process that takes place through the complementary action of osteoclasts and osteoblasts. Osteoclasts break down and resorb old bone while osteoblasts deposit new bone in place; thus, both cell types play key physiological roles [1].

Osteoporosis is a bone disease characterized by reduced low bone mass and microarchitectural deterioration of bone tissue with a consequent increase in bone fragility and susceptibility to fracture, as its most clinically significant aspects [2]. The impact of osteoporosis on quality of life is expected to increase as the elderly population continues to grow [3].

All established osteoporosis therapies, such as the use of bisphosphonates, selective estrogen-receptor modulators and denosumab, mainly inhibit bone resorption, but they do not induce bone formation. The search for bone-building (anabolic) agents with the capacity to reduce fracture risk to a greater extent than antiresorptive drugs led to the discovery of teriparatide [4]. Teriparatide is the biologically active fragment of the human parathyroid hormone (PTH), consisting of its first N-terminal 34 amino acids (AAs). PTH plays an important role in calcium and phosphate homeostasis. The type1 PTH receptor (PTH1R) binds both PTH and teriparatide and belongs to the G protein-coupled receptor (GPCR) family. Ligand binding to this receptor activates both the adenyl cyclase and phospholipase C systems, inducing signaling cascades through protein kinases A (via cyclic adenosine monophosphate (cAMP)) and C [5].

*E. coli* produced the recombinant Teriparatide Eli Lilly (from now on referred to as reference medicinal product, RMP) as the first anabolic drug approved in the US (2002) [4] and in the EU (2003) [6] for the treatment of osteoporosis. A daily administration increases the apposition of new bone on trabecular and cortical bone surfaces by preferential stimulation of osteoblastic activity over osteoclastic activity, thus increasing bone density [7]. Teriparatide is formulated as an aqueous solution containing a 0.025% *w*/*v* of teriparatide (20 µg/80 µL/dose) in acetate buffer at pH 4 and preserved with 0.3% *w*/*v* metacresol. It is presented in a 3 mL cartridge assembled into a disposable product-dedicated pen injector that contains treatment for 28 days.

Teriparatide can also be chemically synthesized by solid-phase peptide synthesis (SPPS), a method that has remarkably improved in recent decades, becoming pivotal in modern peptide production. Compared with recombinant technology, the crude peptides obtained by SPPS are more monotonous and free from biological origin compounds such as enzymes, DNA and RNA fragments, non-related proteins and peptides. In addition, the purification of the final SPPS product is relatively straightforward [8]. Nowadays, SPPS efficiently and robustly produces highly purified synthetic peptides. In fact, recombinant and synthetic teriparatide versions co-exist.

Teriparatide BGW (BCN Peptides, S.A.; GP-Pharm, S.A.; Welding GmbH and Co. KG) is the first generic equivalent to the recombinant RMP teriparatide. The product was authorized in the EU in 2020. In addition to the requirements of a generic drug [9], teriparatide BGW has demonstrated, with an extensive side-by-side comparative exercise, a structural, purity profile and biological activity similar to the recombinant teriparatide in the RMP (Table 1).

Here, we evaluated the structural and biological similarity of teriparatide BGW and the RMP. In this regard, we specifically used nuclear magnetic resonance (NMR) and chemometric principal component analysis (PCA) methodology to characterize the peptide and excipient compositions of the two formulations, as well as the higher-order structure (HOS) and conformers of teriparatide in each formulation. Furthermore, we share a practical approach (i.e., ion mobility mass spectrometry, IM–MS) for comparing not only the primary structure and corresponding mass charge ratio of peptides, but also their shape and size in their native state in the formulation. Overall, we used a wide set of orthogonal techniques to prove that synthetic and recombinant peptides behave in the same manner. Finally, we present functional comparative data obtained from two in vitro cell-based bioassays to confirm the bio-similarity.

## 2. Materials and Methods

### 2.1. Drug Products

Several batches of Teriparatide BGW and RMP, at different stages of product shelf life, were used to perform the comparative studies.

### 2.2. Peptide and Excipient Composition Assessment

#### 1D-NMR

Sample preparation: 1D-NMR experiments were performed in native conditions, with the original formulations spiked with 10% D_2_O.

NMR equipment: NMR spectra were acquired using a Bruker Avance III 600 spectrometer working at 600.23 MHz (^1^H frequency), equipped with a z-pulse field gradient unit and a triple (^1^H, ^13^C, ^15^N) resonance probe head. Structures were calculated using a computer grid running the Crystallography and NMR System (CNS) package. The 1D-1H experiments for all samples were performed using standard pulse programs including pre-saturation or a Watergate pulse sequence, to suppress the solvent (H_2_O). These pulse programs were provided by the spectrometer manufacturer (Bruker BioSpin GmbH, Rheinstetten, Germany). Experiments were performed with 32,768 points and 0.21 s detection time.

After Fourier transformation, the data were phase-corrected and additional baseline corrections were applied equally to all datasets. For this purpose, the TOPSPIN program was used (provided by the spectrometer manufacturer Bruker). Binning was performed using the average sum of the spectra, with a bin width of 0.01 ppm. Spectra were normalized using the total spectral sum, and weighting was automatically scaled using the Pareto method.

The PCA was carried out using the software MestReNova 11.0. Statistical analysis was performed by using Multivariate ANOVA (R 4.1.0) and MATLAB Mahalanobis function [10] was applied to calculate the Mahalanobis distance (DM).

### 2.3. High-Order Structure Assessment

#### 2.3.1. 2D-NMR

Sample preparation: 2D-NMR experiments required a partial dialysis of each sample in 90%H_2_O/10%D_2_O overnight to reduce the contribution of excipient resonances to the NMR spectra.

NMR data: 2D-^1^H TOCSY experiments were recorded using DIPSI2 sequence for the magnetization transfer, (50 ms mixing time) and a total of 600 fids in the indirect dimension. We performed 32,768 points in the direct dimension, 16 scans per FID with an acquisition time of 0.14 s per FID. The 2D-^1^H NOESY experiments were recorded using 140 ms mixing times and a total of 600 fids in the indirect dimension. We performed 32 scans per FID with an acquisition time of 0.14 s per FID. CNS software (version 2) was used to determine peptide structure in solution. The 2D ^13^C-^1^H HSQC (Heteronuclear Single-Quantum Correlation) experiments were performed with 2 K and 128 points in the direct and indirect dimensions, respectively, with 256 experiments per FID and echo-anti-echo selection with the carrier centered at 40 ppm.

Structures were calculated using a computer grid running the CNS package.

#### 2.3.2. UV–Vis

Sample preparation: Samples were analyzed in their original formulation.

UV equipment: A NanoDrop OneC (Thermo Fisher Scientific Inc., Waltham, MA, USA) high resolution UV–Vis spectrophotometer provided with a software capable of suppressing excipient interference was used for the analysis. M-cresol and acetate buffer UV spectra were used for the proper subtraction of the excipients’ contributions. Samples were measured as triplicates from 350 to 220 nm in intervals of 0.5 nm. One µL solution drops was directly applied to the pedestal, as recommended by the manufacturer. Second derivative spectra were calculated using the Savitzky–Golay algorithm with a five-point data filter with UVProbe 2.62 software (Shimadzu, Kyoto, Japan) application from 310 to 250 nm.

#### 2.3.3. IM–MS

Sample preparation: Original formulation samples were diluted 1/5 in 200 mM ammonium acetate and then directly injected for MS analysis.

IM–MS equipment: IM–MS experiments were performed using a Synapt G1-High Definition Mass Spectrometer (Waters, Manchester, UK). Samples were infused by automated chip-based nanoelectrospray using a Triversa Nanomate system (Advion BioSciences, Ithaca, NY, USA) as the interface. The ionization was performed in positive mode using a spray voltage and a gas pressure of 1.75 kV and 0.5 psi, respectively. The source pumping speed in the backing region (5.56 mbar) of the mass spectrometer was reduced to achieve optimal transmission of non-covalent complexes. Cone voltage, extraction cone and source temperature were set to 45 V, 3 V and 40 °C, respectively. Trap and transfer collision energies were set to 6 V and 4 V, respectively. The pressure in the Trap and Transfer T-Wave regions were 2.44 × 10^−2^ mbar of Ar and the pressure in the ion mobility spectrometry (IMS) T-Wave was 0.478 mbar of N_2_. Trap gas and IMS gas flows were 8 and 25 mL/s, respectively. The travelling wave used in the IMS T-Wave for mobility separation was operated at a velocity of 300 m/s. The wave amplitude was fixed to 8 V. The bias voltage for entering the T-wave cell was 15 V. The instrument was calibrated over the *m*/*z* range of 500–8000 Da using a solution of cesium iodide. MassLynx version 4.1 SCN 704 and Drift scope version 2.4 software were used for data processing. Experimental drift times were transformed into collision cross-sections (CCS, Å2) by constructing a calibration curve with tryptic peptides of known CCSs [11].

GraphPad software v. 6.01 was used to process IM time distributions mirror plot.

#### 2.3.4. Circular Dichroism (CD) and Fourier Transform Infrared (FTIR)

Sample preparation: Teriparatide was extracted from the formulation matrix by using resin cartridges (CD samples) or by HPLC collection with an acetonitrile–water system (FTIR samples). It was then lyophilized and dissolved in water at the concentration of the final product (0.25 mg/mL).

CD equipment: Extracted teriparatide samples were dissolved in water at the concentration of the final drug product (0.25 mg/mL) and characterized by CD spectroscopy in the far-UV spectral region (190–260 nm). CD spectra of samples were measured using a 0.1 cm path-length cell from 260 to 190 nm in a Jasco J-815 spectropolarimeter and resulted in an average of 4 scans in two different matrices, namely in water at 0.25 mg/mL and in a water–trifluoroethanol (TFE) 1:1 mixture at 0.125 mg/mL. The recorded spectra were deconvoluted by using the BeStSel software (https://bestsel.elte.hu/contact.php; accessed: 25 June 2020).

FTIR equipment: FTIR spectra from extracted teriparatide samples were recorded with a Brucker spectrometer ALPHA II (Brucker) equipped with a platinum attenuated total reflectance (ATR) module from 4000 to 400 cm^−1^ under a resolution of 2 cm^−1^ and 256 scans per sample. FTIR data were analyzed by converting the spectrum to second derivative spectra using the Savitzky–Golay function, smoothed up to 9 points and normalized by means of OPUS software (version 6.5). Estimation of secondary structure was performed with the QUANT2 package.

### 2.4. Biological Activity Assessment

#### 2.4.1. SaOS-2- PTH1R- Homogeneous Time Resolved Fluorescence (HTRF)

Sample preparation: Several concentrations were tested in triplicate. For the preparation of the different concentrations, the original formulation was diluted with vehicle. Vehicle composition contained 0.41 mg of glacial acetic acid and 0.10 mg of sodium acetate per 1 mL of water, adjusted to pH = 4 when necessary, with NaOH 0.1 M or HCl 0.1 M.

Method: The PTH1 Human Parathyroid Hormone GPCR Cell-Based Agonist cAMP Assay (Item 2260, Eurofins Cerep) was used to compare the PTH1 agonist response of Teriparatide BGW and RMP samples in SaOS-2 cells endogenously expressing PTH1R and determined by HTRF technology. The EC_50_ values (concentration producing a half-maximal response) were determined by non-linear regression analysis of the concentration–response curves generated with mean replicate values. This analysis was performed using software developed at Cerep (Hill software) and validated by comparison with data generated by the commercial software SigmaPlot^®^ 4.0 for Windows^®^ (©1997 by SPSS Inc., Chicago, IL, USA). After qualification with PTH (1-34) standard reference agonist, the assay acceptance range was set as mean EC_50_ ± ½ log.

#### 2.4.2. CHO-K1- PTH1R- Enzyme Fragment Complementation (EFC)

Sample preparation: Several concentrations were tested in duplicate.

Method: The PTH1 Human Parathyroid Hormone GPCR Cell-Based Agonist cAMP Assay (DiscoverX) was used to compare the PTH1 agonist response of teriparatide BGW and RMP samples in CHO-K1 cells overexpressing PTH1R and determined by EFC technology. Compound activity was analyzed using the CBIS data analysis suite (ChemInnovation, San Diego, CA, USA). After qualification with PTH (1-34) standard reference agonist, the assay acceptance range was set as mean pEC50 of 9.66 ± 0.88.

## 3. Results and Discussion

### 3.1. Comparative Analysis of the Peptide and Excipient Composition Assessment

#### 1D-NMR + PCA

Chemometric methods (principal component analysis, PCA) were applied to compare the two compositions by 1D-NMR. This methodology allows for the comparison of complex biological samples in a systematic manner [12], since NMR provides fingerprint (proton or carbon) information of the molecules present in the formulations (peptides and excipients). In this way, full formulations can be compared, and the similarities and differences can be quantified. All chemometric methods lie in the construction of common factors (or principal components, PCs) that define the original data by reducing multivariate data into a few dimensions that can be graphically displayed.

To perform the PCA, we defined two regions in the spectra: from 8.0 to 5.0 ppm and from 4.0 to 1.5 ppm. These regions contain resonances from the samples and excipients while excluding the water signal region (from 4–5 ppm), which is perturbed by the water suppression protocol used to acquire the 1D-NMR data.

**Figure 1:**
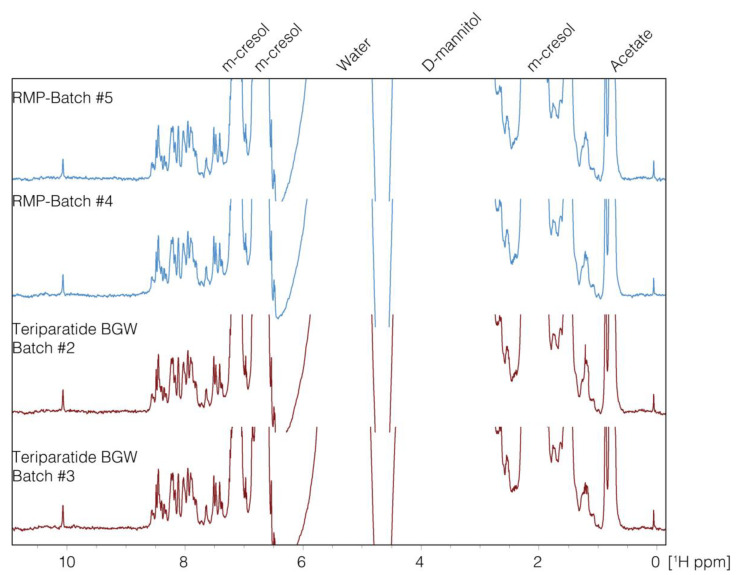
1D-NMR spectra (8.0–1.0 ppm). Four datasets of different batches are shown for comparison: two samples correspond to Teriparatide BGW Batch #2 and #3 and two correspond to reference medicinal product (RMP) Batch #4 and #5.

**Figure 2:**
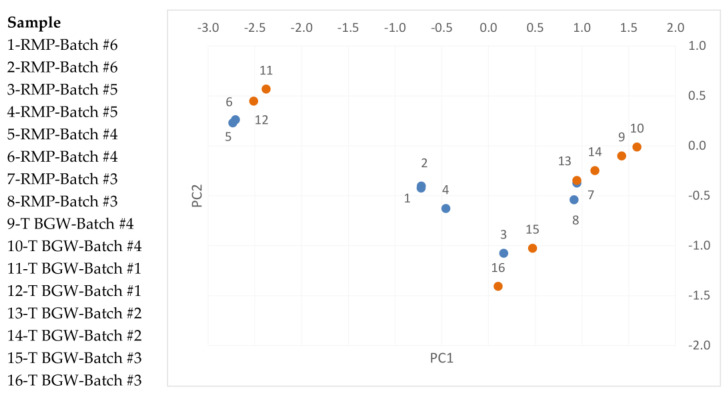
Chemometrics analysis; Principal components (PCs) 1 vs. PC2 plot. PC1 and PC2 values are represented in the X and Y axis, respectively. The data distribution indicates that the two sets of formulations cannot be separated by PC values (*p* = 0.37), indicating the sameness of the samples. RMP (Batch #3–#6) PC values (#1–8) are depicted in blue; Teriparatide (T) BGW (Batch #1–#4) PC values (#9–16) are depicted in orange.

The region (8.0–1.0 ppm) of the NMR 1D spectra displaying the resonances of both teriparatide BGW and RMP formulations is shown in Figure 1. We observed that PC1 described 59.9% of the formulations and PC2 24.0%, both accounting for 83.9% of the formulations. After plotting PC1 vs. PC2 values (Figure 2), the distribution data of teriparatide BGW and the RMP clustered, indicating that the samples of teriparatide BGW and the RMP are non-distinguishable by PC values (*p* = 0.37), thereby confirming their sameness in terms of peptide, excipients and formulation properties.

Furthermore, since the PC1 and PC2 values described more than 80% of the data, these two coordinates were used for the DM calculations. DM is a unitless and scale-invariant distance used, amongst other applications, in analytical similarity assessments to compare two presumably identical formulations. Depending on the system analyzed, DM values can vary from 0 (fully identical) up to several hundreds. For example, the value reported in the original paper describing the methodology [10] was 213 for two samples on the market assumed to be of equal composition. Thus, the DM value (0.57) obtained for the comparison between Teriparatide BGW and the RMP samples corroborated the sameness of the two formulations in terms of composition.

### 3.2. High-Order Structure Assessment

#### 3.2.1. 2D-NMR

To further illustrate the sameness between the formulations of teriparatide BGW and the RMP, 2D 1H NMR (2D NOESY and 2D TOCSY) spectroscopy experiments were performed with one batch of each formulation. These experiments allowed for the assignation of the resonances corresponding to the peptide as well as the medium and long-range interactions between residues, and the determination of the three-dimensional structure of the peptide in the formulations.

As deduced from the PCA analysis, the superimposition of 2D TOCSY experiments corroborated the sameness of the two samples, since the superimposition of all resonances indicates that they are identical, thereby allowing us to conclude that all AAs in the two samples adopt the L configuration and also that the secondary and tertiary properties are also identical (Supplementary Information, Figure S1).

**Figure 3:**
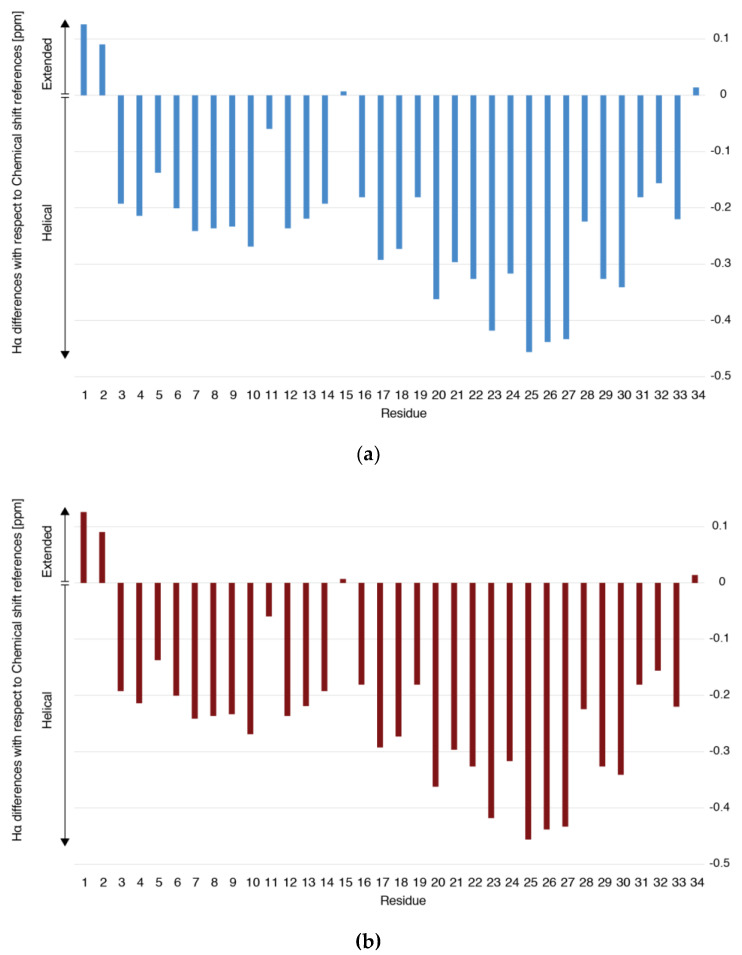
Graphical representation of Hα differences with respect to chemical shift reference. (**a**) RMP, Batch #6; (**b**) Teriparatide BGW, Batch #2.

In fact, NOEs that define the presence of secondary structural elements were assigned, such as Hαi/HNi + 1, HNi/HNi + 1 and HNi/HNi + 3, confirming that recombinant and synthetic peptides adopt helical conformations in the formulations. For instance, the intensity of the HNi/HNi + 1 NOEs is larger than that of the Hαi/HNi + 1 counterpart, indicating that the peptide dissolved in this solution (mannitol, meta-cresol and acetic acid) populates an ensemble of helical conformations. The pattern of NOEs (Hαi/HNi + 3 and HNi/HNi + 1) as well as the differences observed in the Hα chemical shifts compared to that of the reference AAs corroborate the presence of two short helices, comprising residues Ser3-Gly12 and Glu19- Lys27 in both samples. As observed in Figure 3, the C-terminal part has a higher helical propensity than the N-terminal one. In addition to these correlations, long-range NOEs were detected between the aromatic ring of Trp23 and the methyl groups of Leu15. These long-range NOEs induce a short turn that connects the helical pair.

**Figure 4:**
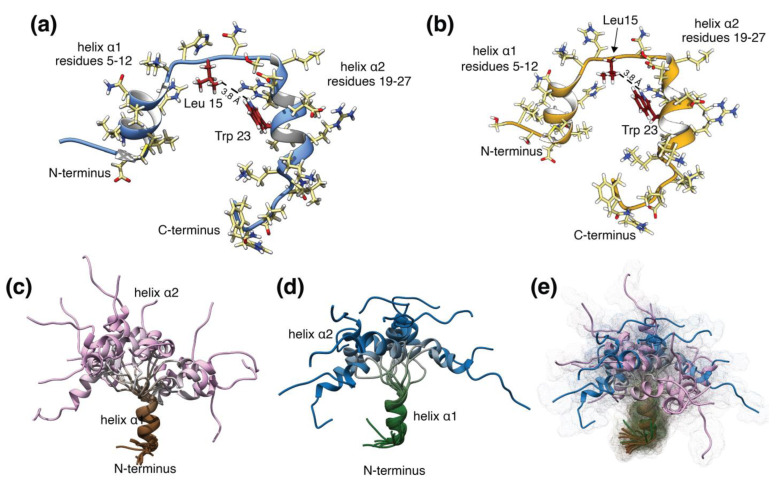
Teriparatide minimal energy structures and NMR structural ensembles. (**a**) Minimum energy structure of the RMP, Batch #6; (**b**) Minimum energy structure of teriparatide BGW, Batch #2; (**c**) NMR structural ensembles for RMP, Batch #6; (**d**) NMR structural ensembles for teriparatide BGW, Batch #2; (**e**) Conformational space explored by both ensembles, represented as a dotted 3D surface.

The restraints corresponding to the presence of the helices and the few long-range NOEs between Trp23 and Leu15 were used to determine the peptide structure in solution using the CNS software, which is commonly used for macromolecular structure determination using X-ray or solution NMR spectroscopy data [13]. Even with these atomic contacts, the structure shows flexibility, as expected for a medium-sized peptide, since these NOEs do not restrict the orientation of the helices with respect to one another and different relative orientations of the helices can satisfy this restraint and the remaining NOEs. This feature is highlighted in Figure 4, where one helix was used as the reference to superimpose the ensemble of conformations adopted for the peptide in solution. The observed conformations are very similar to those previously described in aqueous solution for teriparatide peptides and for the studies in phosphate-buffered solution in the literature, also determined by NMR [14].

Overall, the 2D-NMR analyses of these samples (secondary and tertiary structure in the final preparation) confirm the results obtained using the 1D-NMR-PCA analysis and support the sameness between teriparatide BGW and the RMP.

#### 3.2.2. UV

**Figure 5:**
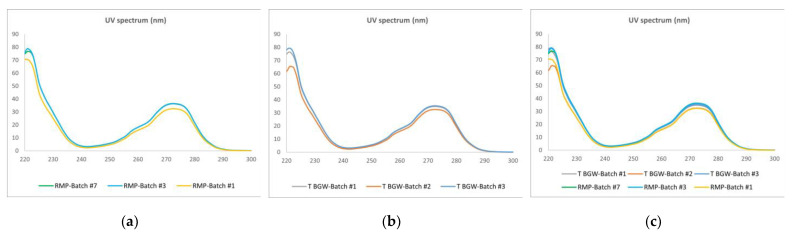
UV–vis absorbance spectra. (**a**) RMP, Batch #1, #3 and #7; (**b**) Teriparatide BGW, Batch #1, #2 and #3; (**c**) Overlay of RMP and teriparatide BGW batches. Absorbance values on the y-axis are expressed in arbitrary units.

**Figure 6:**
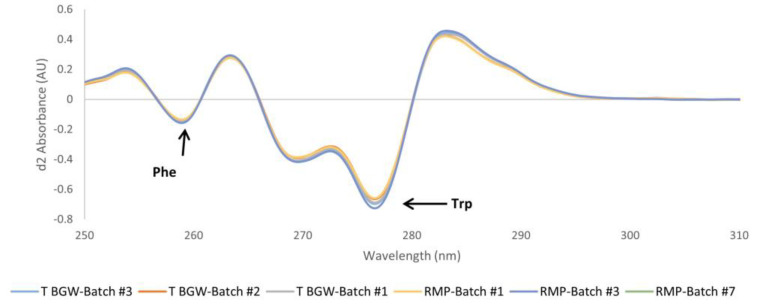
Superposition of the second derivative UV spectra of teriparatide BGW, Batch #1, #2 and #3 and RMP, Batch #1, #3 and #7.

We also studied the identity of high-order structures for the two formulations using UV spectrometry. As shown in Figure 5, the peptide contribution is centered around 270 nm and is identical in all samples. Further analysis, by calculating the second derivative spectra in the near-UV region (250–310 nm), also showed identical profiles among the different batches of teriparatide BGW and its RMP (Figure 6), thereby indicating the similarity of the microenvironment of the aromatic residues Phe and Trp within the teriparatide sequence in both products. Therefore, the superposition of the UV zero-order and UV second derivative spectral profiles of the two products confirms their sameness in secondary and tertiary structure conformation.

#### 3.2.3. IM–MS

IM–MS data were also acquired for teriparatide BGW and the RMP, because this coupled technique allows for the simultaneous comparison not only of their mass to charge ratio, but also of their shape and size. IM–MS was used to analyze the structure and conformation of intact teriparatide ions in their native state. IM rapidly separates ions (microseconds to milliseconds) based on their mobility in a gas under the influence of an electric field, which primarily depends on ion shape (collision cross-section value, Ω) and charge (z). Collision cross section (CCS) measurements in a travelling wave ion mobility system (TWIMS) [15,16] were used to study the gas phase conformers of teriparatide from teriparatide BGW and the RMP.

**Figure 7:**
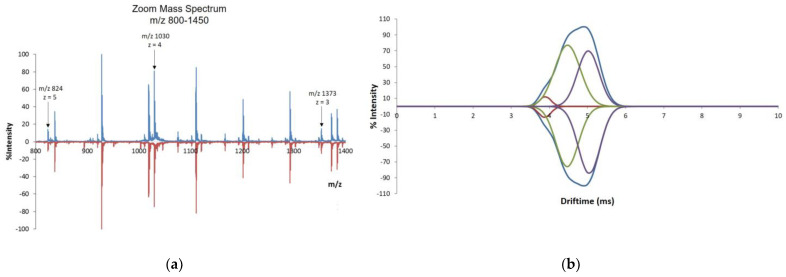
IM–MS mirror comparison. (**a**) Mirror plot of mass spectrum from one batch of teriparatide BGW (batch #1, top) and one batch of RMP (batch #5, bottom); (**b**) Mirror plot comparison of ion mobility time distributions for the selected *m*/*z* 1030 (*z* = 4) of teriparatide from one batch of teriparatide BGW (batch #1, top) and one batch of RMP (batch #5, bottom). Red, green, and purple lines correspond to the three types of peptides or conformers for ion m/z 1030 (z=4) separated by IM-MS with collisional cross sections C1, C2 and C3 respectively, both in teriparatide BGW and in RMP. Blue line is the sum of the three conformers.

In a first evaluation, a mirror comparison of the mass spectra obtained for teriparatide BGW and RMP batches showed a high degree of similarity, as observed in the example of Figure 7. A mirror comparison of the ion mobility time distributions (drift time) of the conformers characterizing chemically synthesized teriparatide in teriparatide BGW and RMP is also shown in Figure 7.

The cross-section of an ion is a measure of its overall shape and is thus related to its structure. Experimental CCS (Å2) measurements of the conformations of ion *m*/*z* 1030 (*z* = 4) were determined (Table 2). The mean CSS (A2) values for each of the three conformers (C1, C2 and C3) of teriparatide BGW and the RMP formulations were calculated and compared. The statistical analysis (*t*-test) confirmed that there is no statistically significant difference between the three conformers present in the two formulations (*p* > 0.05): conformer 1 (C1) mean CCS (Å2) teriparatide BGW = 623.0 vs. RMP= 627.1; conformer 2 (C2) mean CCS (Å2) teriparatide BGW = 671.7 vs. RMP = 674.8; and conformer 3 (C3) mean CCS (Å2) teriparatide BGW = 710.7 vs. RMP = 717.

IM–MS separates peptide ions on the basis of differing cross-sections and molecular charge. Therefore, it is an added structural measurement to characterize ions with the same m/z.. As a characteristic of different ions, peptide ion drift time can be used to enhance confidence in protein identifications [17]. Ion mobility separation detected three distinct ion types for teriparatide with similar cross-section areas to those of the RMP, thereby confirming the sameness in charge, size and shape of the different batches of teriparatide BGW and the RMP. These two products show the same type and distribution conformations, thus confirming their similarity in secondary and tertiary structures.

#### 3.2.4. FTIR and CD

The results obtained by NMR, UV and IM–MS confirm the structural similarity of Teriparatide BGW and the RMP, since these studies provide results based on the direct comparison of the drug products (native sample comparison).

To complete the picture, spectroscopic analyses by FTIR and CD were conducted. Our results provide complementary information about the comparability between the synthetic and recombinant teriparatide and thus of the formulations, even if these analyses imply sample manipulation (teriparatide extraction).

**Figure 8:**
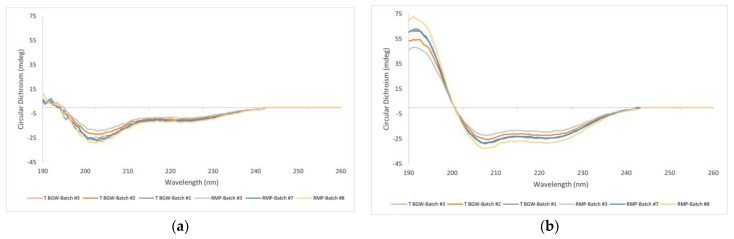
CD spectra of teriparatide. (**a**) Teriparatide extracted from product matrix in water media; (**b**) Teriparatide extracted from product matrix in water–trifluoroethanol (TFE) (1:1) media. Teriparatide BGW, Batch #1, #2 and #3; RMP, Batch #3, #7 and #8.

The CD spectra of the samples were measured to test the α-helix structure propensity of synthetic and recombinant teriparatide (Figure 8), and the recorded spectra were deconvoluted to calculate the ratio of the second structural elements (Table 3). In aqueous solution, teriparatide is mainly disordered, with an average helix content of 10–16%, but when TFE is added, an α -helical structure is induced in both samples. These results are in agreement with published data [18,19] that describe a higher disordered molecule in water and an α-helix ratio content of 40% or higher in the presence of 50% TFE.

**Figure 9:**
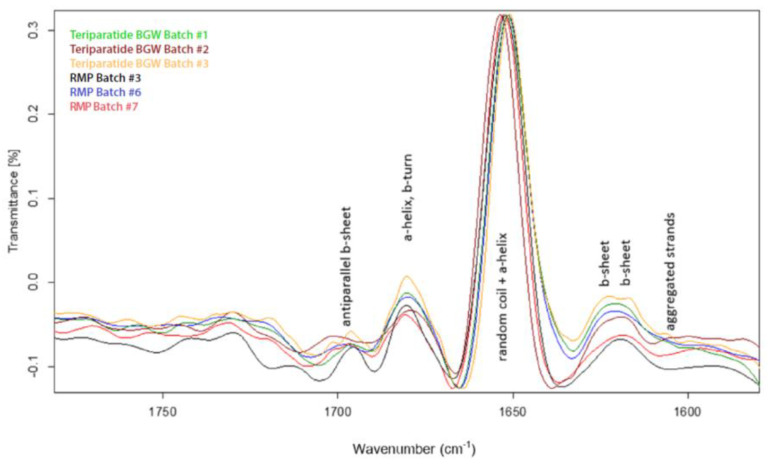
Second derivative FTIR spectra of teriparatide extracted from product matrix. Amide I region FTIR spectra (1900–1300 cm^−1^). Teriparatide BGW, Batch #1, #2 and #3; RMP, Batch #3, #7 and #8.

In addition, we evaluated the secondary structures of teriparatide using FTIR spectroscopy. Comparison of second derivative FTIR spectra for synthetic and recombinant teriparatide active pharmaceutical ingredients (APIs) in the Amide I region is shown in Figure 9, substantiating that teriparatide BGW and the RMP are equivalent in terms of teriparatide secondary structure.

The second derivative spectra peaks obtained were assigned to the α-helix, β-sheets and β-turns and random secondary structures. Moreover, a PLS-algorithm (Opus) was used to estimate the percentage of α-helix and β-sheet. The results predict an average content of 50% of α -helix and 16% of β -sheet for teriparatide peptide extracted from the two formulations. The obtained results are consistent with those reported in the literature about secondary structure conformation of teriparatide in solid form [21] and confirm the similarity between the secondary structures of teriparatide BGW and RMP drug products.

### 3.3. Biological Activity Assessment

As demonstrated above, teriparatide BGW and the RMP present structural and conformational sameness. Therefore, both products are expected to have the same biological activity, as confirmed by the following in vitro assays.

In osteoblasts, PTH acts via PTH1R, which activates several signal transduction pathways [22]. Among these pathways, cAMP is the dominant mechanism that drives the bone anabolic effect [23,24,25,26]. This observation forms the basis of the functional cellular assays for the assessment of PTH variants potency based on intracellular cAMP quantification [22].

#### 3.3.1. SaOS-2-PTH1R-HTRF

**Figure 10:**
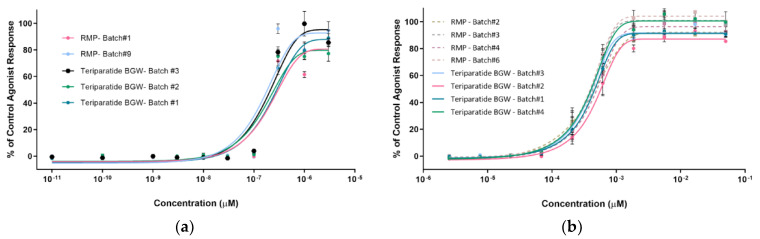
PTH1 agonist response comparison. (**a**) SaOS-2- PTH1R-HTRF assay. Teriparatide BGW, Batch #1, #2 and #3; RMP, Batch #1 and #9.; (**b**) CHO-K1- PTH1R- EFC assay. Teriparatide BGW, Batch #1, #2, #3 and #4.; RMP, Batch #2, #3, #4 and #6.

The first assay was performed with human osteosarcoma cells SaOS-2, which belong to the same linage of osteoblasts, the target cells of teriparatide. In fact, SaOS-2 cells display several osteoblastic features. They endogenously express PTH1R and produce cAMP in response to PTH treatment. The PTH1 agonist response obtained for the different teriparatide BGW and RMP batches is shown in Figure 10. The pEC_50_ values for RMP batch#1 and #9 (pEC_50_ = 6.5M and 6.6M, respectively) and teriparatide BGW batch #1, #2 and #3 (pEC_50_ = 6.6M, 6.8M and 6.7M, respectively) are within the acceptance range (mean EC_50_ ± ½ log), thereby indicating that the five formulations present a similar agonist response.

#### 3.3.2. CHO-K1-PTH1R-EFC

The results obtained in the first assay were confirmed in a second assay, using a non-osteogenic cellular context. This assay was performed with a CHO-K1 cell line, in which human PTH1R was overexpressed. Four batches of RMP (batch #2, #3, #4 and #6) and four batches of teriparatide BGW (batch #1–#4) were evaluated.

As shown in Table 4 and in Figure 10, potency results based on cAMP production confirmed the similarity of teriparatide BGW and the RMP formulations in non-osteogenic cells overexpressing human PTH1R. Furthermore, all results are within the range of 60–120% of the relative potency to RMP formulations (mean: 99%, range of 86%–119%), fulfilling the acceptance criteria described in the USP current edition monograph for teriparatide.

Notably, an expired RMP sample (batch #2) with 93% of purity but not complying with purity specifications in terms of PTH(1–30), showed a relative potency within the acceptance criteria. Thus, as previously described [27,28], teriparatide peptide-related impurities appearing along the shelf life are also active but with varying potencies.

### 3.4. Discussion

Teriparatide BGW and the RMP have the same qualitative and quantitative composition in terms of drug substance and excipients, as well as the same pharmaceutical form. Based on the extensive comparisons using a wide range of state-of-the-art orthogonal methods, the similarity of synthetic teriparatide in teriparatide BGW and recombinant teriparatide in the RMP has been demonstrated.

The teriparatide BGW regulatory procedure was discussed with the relevant regulatory agencies, the generic application being the optimal approach as it fulfils the eligibility criteria established in the EU legislation for a generic application [9]. Hence, teriparatide BGW is the first generic teriparatide approved in the EU (2020).

The choice of the generic pathway was reinforced by the release, a year after, of a specific FDA guideline [29]. This guidance helps sponsors bring highly purified synthetic peptides, such as teriparatide, to market as complex generic drugs. With the advances in analytical methods, the FDA and European Agencies recognize that it is now possible to demonstrate that the active ingredient in a proposed generic synthetic peptide is the same as that of the RMP. Sameness demonstration involves, in addition to the general requirements for a generic drug, a stepwise comparison from primary sequence and physicochemical properties to HOS and biological activity, which is accomplished by teriparatide BGW. The FDA guideline also establishes purity constraints that should be fulfilled by the generic drug product, which are also accomplished by teriparatide BGW.

Since the RMP’s patent expiration in 2019, other teriparatides have been marketed in the EU. We unequivocally demonstrated that the synthetic peptide of teriparatide BGW is structurally the same as the recombinant peptide of the RMP. Therefore, according to the EU bioequivalence guidelines in force [30], a bioequivalence study was not necessary. Should this have not been the case, appropriate preclinical and clinical studies may have been required to prove the equivalent efficacy and safety profile compared to the RMP [31,32].

One of the advantages of synthetic over recombinant peptide lies in the impurity profile. Specifically, synthetic and recombinant peptide products may contain product-related and process-related impurities. The former are derived from the main peptide ingredient and are common for recombinant and synthetic peptides. The immunogenicity risk of peptides is generally accepted to be negligible in fully human endogenous peptides and peptides of less than 40 AAs [33]. Since teriparatide has less than 40 AAs, all naturally found in human peptides and proteins, an immunogenic response should not be expected. Nevertheless, process-related impurities for recombinant teriparatides may include other product-related impurities (i.e., cell substrates including host cell proteins, host cell DNA, cell culture, among others), which results in a potential risk of immunogenicity [34]. In contrast, teriparatide obtained by chemical synthesis abrogates this risk since no biological source is involved in the manufacturing process.

Most biological drugs for osteoporosis are costly, thus limiting patient access. The incidence of fragility fractures markedly increases with age, particularly in women. Considering life expectancy and birth rate in the EU27+2 countries, the number of elderly individuals above 75 years is likely to increase by 43% and 30% for men and women, respectively, requiring further resource allocation for ageing-associated diseases. In fact, the total direct cost of osteoporosis in the EU27+2 (excluding the value of QALYs lost) was EUR 56.9 billion in 2019, which represents approximately 3.5% of healthcare spending. These figures demonstrate the significant impact of fragility fractures on the healthcare budgets of the current EU countries [3].

A recent systematic review, network meta-analysis and meta-regression analysis of 69 randomized clinical trials, including more than 80,000 patients, concluded that bone anabolic treatments reduces the risk of fractures more than antiresorptive agents [35]. Furthermore, this analysis provided no clinical evidence for restricting the use of anabolic treatment to patients with a very high risk of fractures. According to the authors, the reason for recommending bone anabolic treatments specifically for patients at high risk of fractures is based more on cost considerations than on robust evidence, favoring its use in this group over others. In their opinion, with the introduction of biosimilars and generics of teriparatide at a lower cost, the study data could prompt a review of the current guidelines for earlier use of these agents in the treatment of osteoporosis.

In summary, the availability of complex peptide generics results in higher market competition, which is critical for the long-term sustainability of the healthcare system and health equity. Complex peptide generic products are harder to develop as they require a wide range of state-of-the-art methods, high technical capacity and costly resources to demonstrate similarity to the RMP, thereby limiting their production globally. Teriparatide BGW, the first generic teriparatide, provides an available treatment option for patients with osteoporosis. This novel teriparatide, with proven pharmaceutical quality, and thus the same efficacy and safety profile, ensures the RMP’s clinical benefit with a lower potential risk of immunogenicity.

## Figures and Tables

**Table 1:** Summary of side-by-side comparative analysis exercise between teriparatide BGW and its RMP.

Quality Attribute	Analytical Methods	Study Purpose
Composition and strength		
Peptide and excipients composition	1D-NMR	To determine similarity between formulations by using chemometric methods
Peptide content ^5^	RP-HPLC	To quantify peptide content ^2^
Preservative content (m-cresol) ^5^	RP-HPLC	To quantify m-cresol content
Primary structure ^5^		
Amino acid analysis	2.2.56 EP method and <1052> USP method ^1^	To determine amino acid composition
Peptide mapping	UV- and MS ^1^-based	To identify and verify primary structure
Peptide sequence	2D-NMR (2D-TOCSY)	To confirm peptide sequence
Intact molecular mass	ESI-HRMS	To confirm identity and primary structure
High-order structures		
Secondary and tertiary	2D-NMR (2D TOCSY, 2D NOESY)	To qualitatively and quantitatively determine secondary and tertiary structures
Secondary and tertiary	IM–MS	To determine structure and conformation and non-covalent interactions in native MS
Secondary and tertiary	UV-vis	To determine peptide conformation
Secondary	FTIR ^1^	To identify secondary structure
Secondary	CD ^1^	To identify secondary structure
Purity profile ^5^		
Peptide-related variants	RP-HPLC	To determine charge variant impurities ^2^
Peptide-related variants	RP-HPLC	To determine low MW impurities ^2^
Oligomers/aggregates	SE-HPLC	To determine the presence of oligomers/aggregates
Oligomers/aggregates	SDS-PAGE	To determine the presence of oligomers/aggregates
Product-related impurities	RP-HPLC	To compare impurity profile between formulations ^2^
Degradation profile	RP-HPLC	To compare impurity profile between formulations under extreme conditions (40 °C/75% RH)
Functional characteristics		
Biological activity	SaOS-2 PTH1R cell-based bioassay ^3^	To determine agonist response
Biological activity	CHO-K1 PTH1R cell-based bioassay ^4^	To determine agonist response ^2^

^1^ Analyses conducted with the active pharmaceutical ingredient (API) due to the presence of excipients in the formulation that shielded the signals and data of the peptide. ^2^ The analysis was conducted at different stages of product batches’ shelf life and also on batches analyzed after the expiry date. ^3^ HTRF detection method. ^4^ EFC detection method. ^5^ Analyses not presented but conducted as part of the complete characterization of the generic versus the reference product. Abbreviations: NMR (nuclear magnetic resonance); RP–HPLC (reverse phase–high-pressure liquid chromatography); MS (mass spectrometry); ESI–HRMS (electrospray ionization high-resolution mass spectrometry; FTIR (Fourier transform infrared), CD (circular dichroism); UV (ultraviolet); IM–MS (ion mobility–mass spectrometry); SE–HPLC (size exclusion–high-pressure liquid chromatography); SDS-PAGE (Sodium Dodecyl Sulfate PolyAcrylamide Gel Electrophoresis); CHO-K1-PTH1R (Cell Line from hamster ovary expressing the type 1 PTH receptor); SaOS-2 (human osteosarcoma cell line); HTRF (homogeneous time resolved fluorescence) and EFC (enzyme fragment complementation).

**Table 2:** Experimentally determined collision cross-section (CCS) of ion *m*/*z* 1029.78 (*z* = 4) for teriparatide BGW and RMP batches.

Sample	Specie	Charge State	*m*/*z*	Cross-Section (Power) Å2 *
Teriparatide BGW- Batch #1	[M + 4H]^4+^	4	1029.7811	619 (C1)
	[M + 4H]^4+^	4	1029.7811	666 (C2)
	[M + 4H]^4+^	4	1029.7811	710 (C3)
Teriparatide BGW- Batch #2	[M + 4H]^4+^	4	1029.7811	626 (C1)
	[M + 4H]^4+^	4	1029.7811	675 (C2)
	[M + 4H]^4+^	4	1029.7811	716 (C3)
Teriparatide BGW- Batch #3	[M + 4H]^4+^	4	1029.7811	619 (C1)
	[M + 4H]^4+^	4	1029.7811	666(C2)
	[M + 4H]^4+^	4	1029.7811	699 (C3)
Teriparatide BGW- Batch #4	[M + 4H]^4+^	4	1029.7811	628 (C1)
	[M + 4H]^4+^	4	1029.7811	680 (C2)
	[M + 4H]^4+^	4	1029.7811	718 (C3)
RMP- Batch #1	[M + 4H]^4+^	4	1029.7811	628 (C1)
	[M + 4H]^4+^	4	1029.7811	675 (C2)
	[M + 4H]^4+^	4	1029.7811	720 (C3)
RMP- Batch #2	[M + 4H]^4+^	4	1029.7811	627 (C1)
	[M + 4H]^4+^	4	1029.7811	676 (C2)
	[M + 4H]^4+^	4	1029.7811	720 (C3)
RMP- Batch #3	[M + 4H]^4+^	4	1029.781125	630 (C1)
	[M + 4H]^4+^	4	1029.781125	674 (C2)
	[M + 4H]^4+^	4	1029.7811	711 (C3)
RMP- Batch #4	[M + 4H]^4+^	4	1029.781125	628 (C1)
	[M + 4H]^4+^	4	1029.781125	674 (C2)
	[M + 4H]^4+^	4	1029.781125	720 (C3)
RMP- Batch #5	[M + 4H]^4+^	4	1029.781125	625 (C1)
	[M + 4H]^4+^	4	1029.781125	674 (C2)
	[M + 4H]^4+^	4	1029.781125	718 (C3)
RMP- Batch #6	[M + 4H]^4+^	4	1029.781125	625 (C1)
	[M + 4H]^4+^	4	1029.781125	674 (C2)
	[M + 4H]^4+^	4	1029.781125	713 (C3)

* Experimental cross-sections (CCS, Å2) of the conformations of ion *m*/*z* 1030 (*z* = 4) were determined by constructing a calibration curve with tryptic peptides of known CCSs [11].

**Table 3:** CD Spectra. Percentages of each main secondary structural elements predicted by BeStSel algorithm [20] in water and water: TFE (1:1) media.

		BeStSel—Secondary Structure Prediction— Water				BeStSel—Secondary Structure Prediction— Water: TFE (1:1)			
Sample	Batch	α-helix (%)	β-sheet (%)	Turn (%)	Others (%)	α-helix (%)	β-sheet (%)	Turn (%)	Others (%)
Teriparatide BGW	#1	13.7	20.4	17.7	48.2	61.3	0.0	10.7	28.0
	#2	15.2	20.6	17.7	48.2	57.6	1.1	12.4	28.9
	#3	14.9	19.2	18.3	47.5	61.5	0.0	11.0	27.5
RMP	#3	11.3	26.8	17.0	44.8	51.1	5.3	12.9	30.7
	#7	15.3	20.6	17.9	46.2	62.1	0.0	11.4	26.5
	#8	15.8	17.3	18.4	48.5	68.6	0.0	9.1	22.4

**Table 4:** PTH1 agonist response comparison. CHO-K1- PTH1R- EFC assay. EC_50_ determination.

Sample	Batch	EC_50_ (M)	pEC_50_	Mean EC_50_ (M)	% vs. Mean RMP EC_50_	
Teriparatide BGW	#1	3.93 × 10^−10^	9.41	4.03 × 10^−10^	96%	99%
	#2	4.85 × 10^−10^	9.31		119%	
	#3	3.52 × 10^−10^	9.45		86%	
	#4	3.82 × 10^−10^	9.42		94%	
RMP	#2	3.94 × 10^−10^	9.40	4.08 × 10^−10^	97%	100%
	#3	4.07 × 10^−10^	9.39		100%	
	#4	4.52 × 10^−10^	9.34		111%	
	#6	3.78 × 10^−10^	9.42		93%	

## Data Availability

The data presented in this study are available from the corresponding author upon reasonable request.

## Supplementary Materials

The following supporting information can be downloaded at: https://www.mdpi.com/article/10.3390/pharmaceutics16040537/s1, Figure S1: Chemical Shifts for NMR spectra.
