# Supplementary material for: First Generic Teriparatide: Structural and Biological Sameness to Its Reference Medicinal Product
Source: Pharmaceutics. 2024 Apr 13;16(4):537. doi: 10.3390/pharmaceutics16040537 (PMC11054030; doi:10.3390/pharmaceutics16040537)
Supplement: Supplementary file 1 [file pharmaceutics-16-00537-s001.zip › pharmaceutics-2942147-supplementary.pdf]

SER 1	HN 7.94	HA 4.63	HB2 3.78	HB3 3.76									
VAL 2	HN 8.48	HA 4.01	HB 1.89	QG1 0.83	QG2 0.76	HG11 0.85	HG12 0.79	HG13 0.81	HG21 0.75	HG22 0.80	HG23 0.81		
SER 3	HN 8.37	HA 4.21	HB2 3.71	HB3 3.68									
GLU 4	HN 7.97	HA 4.04	HB2 1.88	HB3 1.84	HG2 2.15	HG3 2.11							
ILE 5	HN 7.89	HA 3.87	HB 1.64	HG21 0.72	HG22 0.69	HG23 0.65	HG12 1.30	HG13 1.00	HD11 0.67	HD12 0.69	HD13 0.67		
GLN 6	HN 8.07	HA 4.01	HB2 1.69	HB3 1.69	HG2 2.18	HG3 2.19	HE21 7.42	HE22 6.84					
LEU 7	HN 8.03	HA 3.99	HB2 1.44	HB3 1.46	HG 1.31	QD1 0.72	QD2 0.70	HD11 0.76	HD12 0.67	HD13 0.75	HD21 0.72	HD22 0.67	HD23 0.70
MET 8	HN 8.24	HA 4.24	HB2 1.82	HB3 1.87	HG2 2.38	HG3 2.09	QE 2.19	HE1 1.91	HE2 1.83	HE3 1.85			
HIS 9	HN 8.22	HA 4.42	HB2 3.07	HB3 3.05	HD2 7.05	HE1 8.40							
ASN 10	HN 8.31	HA 4.42	HB2 2.66	HB3 2.63	HD21 7.54	HD22 6.76							
LEU 11	HN 8.11	HA 4.15	HB2 1.51	HB3 1.54	HG 1.41	QD1 0.70	QD2 0.70	HD11 0.67	HD12 0.72	HD13 0.71	HD21 0.63	HD22 0.64	HD23 0.69
GLY 12	HN 8.21	HA2 3.65											
LYS 13	HN 7.91	HA 4.17	HB2 1.55	HB3 1.50	HG2 1.24	HG3 1.13	HD2 1.40	HD3 1.47	HE2 2.81	HE3 2.79	HZ1 7.42	HZ2 7.34	
HIS 14	HN 8.42	HA 4.50	HB2 3.10	HB3 2.93	HD2 7.13	HE1 8.39							
LEU 15	HN 8.15	HA 4.13	HB2 1.37	HB3 1.31	HG 1.28	QD1 0.56	HD11 0.65	HD12 0.63	HD13 0.66	HD21 0.59	HD22 0.63	HD23 0.59	
ASN 16	HN 8.61	HA 4.58	HB2 2.77	HB3 2.72	HD21 7.48	HD22 6.77							
SER 17	HN 8.25	HA 4.22	HB2 3.82	HB3 3.65									
MET 18	HN 8.24	HA 4.25	HB2 1.94	HB3 1.94	HG2 2.44	HG3 2.45	QE 1.83	HE1 1.86	HE2 1.82	HE3 1.84			
GLU 19	HN 8.05	HA 4.18	HB2 1.85	HB3 1.79	HG2 2.41	HG3 2.27							
ARG 20	HN 8.20	HA 4.02	HB2 1.73	HB3 1.57	HG2 1.43	HG3 1.39	HD2 2.95	HD3 2.97	HE 7.10				
VAL 21	HN 7.81	HA 3.69	HB 1.92	QG1 0.87	QG2 0.68	HG11 0.79	HG12 0.77	HG13 0.76	HG21 0.71	HG22 0.69	HG23 0.67		
GLU 22	HN 8.13	HA 4.00	HB2 1.83	HB3 1.88	HG2 2.10	HG3 2.19							
TRP 23	HN 7.98	HA 4.22	HB2 3.19	HB3 3.12	HD1 7.05	HE3 7.41	HE1 9.98	HZ3 6.95	HZ2 7.26	HH2 7.01			
LEU 24	HN 7.94	HA 3.81	HB2 1.49	HB3 1.51	HG 1.36	QD1 0.70	QD2 0.69	HD11 0.73	HD12 0.67	HD13 0.73	HD21 0.64	HD22 0.63	HD23 0.66
ARG 25	HN 7.76	HA 3.88	HB2 1.65	HB3 1.67	HG2 1.47	HG3 1.39	HD2 2.95	HD3 2.93	HE 7.21				
LYS 26	HN 7.91	HA 3.90	HB2 1.45	HB3 1.10	HG2 1.38	HG3 1.87	HD2 1.22	HD3 1.15	HE2 2.76	HE3 2.64	HZ1 7.33	HZ2 7.34	
LYS 27	HN 7.84	HA 3.90	HB2 1.42	HB3 1.55	HG2 1.18	HG3 1.15	HD2 1.28	HD3 1.26	HE2 2.64	HE3 2.43	HZ1 7.36	HZ2 7.39	
LEU 28	HN 7.90	HA 4.09	HB2 1.50	HB3 1.41	HG 1.37	QD1 0.68	QD2 0.67	HD11 0.65	HD12 0.71	HD13 0.67	HD21 0.66	HD22 0.65	HD23 0.66
GLN 29	HN 7.92	HA 4.02	HB2 1.86	HB3 1.85	HG2 2.21	HG3 2.13	HE21 7.02	HE22 6.79					
ASP 30	HN 8.08	HA 4.45	HB2 2.58	HB3 2.46									
VAL 31	HN 7.76	HA 3.83	HB 1.86	QG1 0.61	HG11 0.70	HG12 0.61	HG13 0.66	HG21 0.63	HG22 0.61	HG23 0.65			
HIS 32	HN 8.44	HA 4.46	HB2 2.95	HB3 2.84	HD2 6.96	HE1 8.38							
ASN 33	HN 8.17	HA 4.51	HB2 2.54	HB3 2.46	HD21 7.30								
PHE 34	HN 7.60	HA 4.71	HB2 2.93	HB3 2.78									

(**a**)

SER 1	HN 7.89	HA 4.60	HB2 3.77	HB3 3.83									
VAL 2	HN 8.50	HA 4.06	HB 1.87	QG1 0.82	QG2 0.80	HG11 0.85	HG12 0.75	HG13 0.78	HG21 0.80	HG22 0.78	HG23 0.74		
SER 3	HN 8.32	HA 4.23	HB2 3.71	HB3 3.70									
GLU 4	HN 7.97	HA 4.11	HB2 1.95	HB3 1.85	HG2 2.23	HG3 2.06							
ILE 5	HN 7.96	HA 3.80	HB 1.69	HG21 0.73	HG22 0.70	HG23 0.71	HG12 1.33	HG13 1.04	HD11 0.66	HD12 0.65	HD13 0.74		
GLN 6	HN 8.07	HA 4.08	HB2 1.64	HB3 1.68	HG2 2.23	HG3 2.17	HE21 7.35	HE22 6.83					
LEU 7	HN 7.99	HA 3.96	HB2 1.49	HB3 1.49	HG 1.32	QD1 0.70	QD2 0.72	HD11 0.67	HD12 0.66	HD13 0.69	HD21 0.65	HD22 0.69	HD23 0.71
MET 8	HN 8.25	HA 4.27	HB2 1.89	HB3 1.81	HG2 2.40	HG3 2.09	QE 2.20	HE1 1.97	HE2 1.88	HE3 1.89			
HIS 9	HN 8.17	HA 4.45	HB2 3.09	HB3 3.00	HD2 7.09	HE1 8.38							
ASN 10	HN 8.29	HA 4.51	HB2 2.68	HB3 2.64	HD21 7.53	HD22 6.83							
LEU 11	HN 8.07	HA 4.08	HB2 1.52	HB3 1.47	HG 1.43	QD1 0.75	QD2 0.66	HD11 0.74	HD12 0.71	HD13 0.76	HD21 0.65	HD22 0.70	HD23 0.67
GLY 12	HN 8.21	HA2 3.64											
LYS 13	HN 7.83	HA 4.13	HB2 1.48	HB3 1.53	HG2 1.17	HG3 1.17	HD2 1.47	HD3 1.46	HE2 2.80	HE3 2.75	HZ1 7.45	HZ2 7.36	
HIS 14	HN 8.42	HA 4.46	HB2 3.02	HB3 2.95	HD2 7.05	HE1 8.42							
LEU 15	HN 8.17	HA 4.21	HB2 1.43	HB3 1.32	HG 1.34	QD1 0.65	HD11 0.65	HD12 0.62	HD13 0.68	HD21 0.65	HD22 0.66	HD23 0.64	
ASN 16	HN 8.58	HA 4.49	HB2 2.71	HB3 2.67	HD21 7.50	HD22 6.85							
SER 17	HN 8.20	HA 4.24	HB2 3.80	HB3 3.75									
MET 18	HN 8.16	HA 4.30	HB2 1.92	HB3 1.86	HG2 2.50	HG3 2.45	QE 1.88	HE1 1.89	HE2 1.86	HE3 1.91			
GLU 19	HN 8.04	HA 4.10	HB2 1.88	HB3 1.85	HG2 2.41	HG3 2.31							
ARG 20	HN 8.13	HA 3.98	HB2 1.73	HB3 1.57	HG2 1.46	HG3 1.43	HD2 2.95	HD3 3.01	HE 7.04				
VAL 21	HN 7.81	HA 3.64	HB 1.96	QG1 0.79	QG2 0.68	HG11 0.86	HG12 0.74	HG13 0.83	HG21 0.73	HG22 0.63	HG23 0.64		
GLU 22	HN 8.13	HA 4.01	HB2 1.88	HB3 1.85	HG2 2.10	HG3 2.15							
TRP 23	HN 8.00	HA 4.24	HB2 3.19	HB3 3.15	HD1 7.07	HE3 7.37	HE1 9.95	HZ3 6.94	HZ2 7.35	HH2 6.98			
LEU 24	HN 7.93	HA 3.83	HB2 1.51	HB3 1.44	HG 1.33	QD1 0.74	QD2 0.65	HD11 0.72	HD12 0.70	HD13 0.71	HD21 0.68	HD22 0.68	HD23 0.63
ARG 25	HN 7.78	HA 3.92	HB2 1.62	HB3 1.71	HG2 1.56	HG3 1.37	HD2 2.95	HD3 3.01	HE 7.25				
LYS 26	HN 7.84	HA 3.88	HB2 1.48	HB3 1.09	HG2 1.33	HG3 1.84	HD2 1.21	HD3 1.18	HE2 2.76	HE3 2.67	HZ1 7.30	HZ2 7.33	
LYS 27	HN 7.77	HA 3.92	HB2 1.46	HB3 1.54	HG2 1.20	HG3 1.20	HD2 1.31	HD3 1.28	HE2 2.67	HE3 2.39	HZ1 7.42	HZ2 7.37	
LEU 28	HN 7.92	HA 4.09	HB2 1.45	HB3 1.49	HG 1.43	QD1 0.74	QD2 0.66	HD11 0.72	HD12 0.68	HD13 0.65	HD21 0.66	HD22 0.64	HD23 0.64
GLN 29	HN 7.92	HA 4.00	HB2 1.92	HB3 1.88	HG2 2.22	HG3 2.05	HE21 7.07	HE22 6.85					
ASP 30	HN 8.04	HA 4.39	HB2 2.55	HB3 2.43									
VAL 31	HN 7.81	HA 3.86	HB 1.87	QG1 0.61	HG11 0.62	HG12 0.63	HG13 0.61	HG21 0.68	HG22 0.68	HG23 0.65			
HIS 32	HN 8.41	HA 4.48	HB2 2.94	HB3 2.87	HD2 7.02	HE1 8.39							
ASN 33	HN 8.19	HA 4.53	HB2 2.53	HB3 2.42	HD21 7.34								
PHE 34	HN 7.59	HA 4.61	HB2 2.91	HB3 2.82									

(**b**)

Figure S1. Chemical Shifts for NMR spectra. (**a**) Teriparatide BGW, Batch #2; (**b**) RMP, Batch #6. The small differences observed are due to the peak picking software used to generate the peak lists, and they all fall in the error range corresponding to the NMR resolution of 1H frequency.
